# Active vs. Passive Thermal Imaging for Helping the Early Detection of Soil-Borne Rot Diseases on Wild Rocket [*Diplotaxis tenuifolia* (L.) D.C.]

**DOI:** 10.3390/plants12081615

**Published:** 2023-04-11

**Authors:** Massimo Rippa, Andrea Pasqualini, Rossella Curcio, Pasquale Mormile, Catello Pane

**Affiliations:** 1 Institute of Applied Sciences and Intelligent System “E. Caianiello” of CNR, 80078 Pozzuoli, NA, Italy; rossella.curcio@isasi.cnr.it (R.C.);; 2 Consiglio per la Ricerca in Agricoltura e l’Analisi dell’Economia Agraria, Centro di Ricerca Orticoltura e Florovivaismo, 84098 Pontecagnano Faiano, SA, Italy; andrea.pasqualini@crea.gov.it (A.P.); catello.pane@crea.gov.it (C.P.)

**Keywords:** infrared imaging, rhizoctonia rot, *Rhizoctonia solani*, *Sclerotinia sclerotiorum*, sclerotinia soft rot, thermography

## Abstract

Cultivation of wild rocket [*Diplotaxis tenuifolia* (L.) D.C.] as a baby-leaf vegetable for the high-convenience food chain is constantly growing due to its nutritional and taste qualities. As is well known, these crops are particularly exposed to soil-borne fungal diseases and need to be effectively protected. At present, wild rocket disease management is performed by using permitted synthetic fungicides or through the application of agro-ecological and biological methods that must be optimized. In this regard, the implementation of innovative digital-based technologies, such as infrared thermography (IT), as supporting systems to decision-making processes is welcome. In this work, leaves belonging to wild rocket plants inoculated with the soil-borne pathogens *Rhizoctonia solani* Kühn and *Sclerotinia sclerotiorum* (Lib.) de Bary were analyzed and monitored by both active and passive thermographic methods and compared with visual detection. A comparison between the thermal analysis carried out in both medium (MWIR)- and long (LWIR)-wave infrared was made and discussed. The results achieved highlight how the monitoring based on the use of IT is promising for carrying out an early detection of the rot diseases induced by the investigated pathogens, allowing their detection in 3–6 days before the canopy is completely wilted. Active thermal imaging has the potential to detect early soil-borne rotting diseases.

## 1. Introduction

Baby-leaf vegetable fresh consumption, especially through the high-convenience packaging chain, is growing steadily both in Europe and in other parts of the world, including more than 22 trade references [1]. In particular, cultivation is strongly developing in several countries of the Mediterranean basin thanks to the particular vocation of the areas invested in cold greenhouses and polytunnels and is driven by a lively market attentive to the consumption of light salads linked to a healthy lifestyle and eating pattern [2]. Italy is the leader in the EU for producing salad vegetables, with approximately 90,000 tons harvested [3]. Among the group of baby-leaf vegetables, the most cultivated species is wild rocket [*Diplotaxis tenuifolia* L. (D.C.)], with approximately 6500 hectares covered [4]. This brassicaceous crop is grown intensively in protected cultivation, starting with high-density precision sowing on parallel-row beds, sprinkling fertirrigation, and the mechanization of all operations, including fresh cutting [5], to be brought directly to the consumer’s table in ready-to-use packages respecting the cold chain. Nutritional (low in calories and high in fibers) and taste (pungency, aroma) features, as well as the adoption of sustainable farming methods (biobased agrotechniques), are in demand and particularly appreciated by the buyer/consumer [6]. The phytosanitary management of the young plants is strategic in this respect. Since the crop is particularly susceptible to soil-borne fungal diseases, which are also favored by the relatively warm and humid microclimate, continuous recropping, and high sowing density, it must be effectively protected against pathogens [7]. Attacks of fungal rot agents such as *Rhizoctonia solani* Kühn (Basidiomycota) and *Sclerotinia sclerotiorum* (Lib.) de Bary (Ascomycota) are becoming increasingly frequent and are seriously compromising the yield and quality of the product. Both fungi are parenchymatous pathogens that can cause germination failure and damping-off in pre- and post-emergence on wild rocket; in more advanced stages, they may evolve, respectively, in tendentially dry [8] and soft [9] rots at the collars and on the leaves. The affected seedlings show stunted growth, yellowing, and, in the most severe cases, premature leaf senescence and death, to the extent that visible blotches may form on the beds. The protection of wild rocket against these two pathogens can be entrusted to the use of synthetic fungicides or, in cases where their use is restricted or prohibited, to the implementation of agro-ecological methods (i.e., the use of suppressive soil amendments, antagonistic microbial consortia, resistance inducers, etc.) in the preventive phase and biologically derived methods (i.e., microorganisms, phytochemicals, etc.) in the curative phase [10,11,12]. However, in the pursuit of objectives to reduce the use of synthetic pesticides, it is necessary to implement innovative strategies that can still increase the effectiveness of alternative control methods and guarantee adequate levels of protection. These latter requests also meet the needs of research in the field of precision agriculture regarding the development of tools able of providing remote and real time information on the physiological status of the plants, including detrimental interactions with different pathogens. Digital technologies, such as infrared thermography (IT), can contribute to this.

IT represents an outstanding innovation able to revolutionize many fields that are gaining great interest in plant studies and in agriculture. It was used on plants in many applications, at both the proximal and remote scales, to evaluate physical and physiological characteristics, such as heat capacity of the leaves, local water content, transpiration rates, water flow velocity, and response to UV interaction, as well as to detect biotic and abiotic stress or foreign substances [13,14,15,16,17,18,19,20,21,22,23,24]. Furthermore, in recent years, several studies have demonstrated how this optoelectronic technology can allow monitoring and predicting plant diseases at an early stage using the spatial distribution of the leaf temperature as an indicator [25]. Recently, among these, IT data were used to identify injuries in guava (*Psidium guajava* L.) during cooling and storage at different temperatures [26], to predict the onset of the disease in wheat (*Triticum aestivum* L.) infected with *Zymoseptoria tritici* (Desm.) [27], and to detect viral diseases in sweet potato (*Ipomoea batatas* L.) [28] and bacterial infection in melon (*Cucumis melo* L.) [29]. Information derived from crop images captured in the mid- to long-term infrared (IR imaging), associated with the gradient of energy emitted by the different parts of the canopy and correlated with the health status, can be used for the monitoring and/or early detection of infection outbreaks and to accurately guide disease management interventions.

In this work, using IT, wild rocket plants inoculated with the soil-borne pathogens *R. solani* and *S. sclerotiorum* were monitored daily by means of both active and passive thermography and compared to control plants. A comparison was made between the results obtained from the two main thermal spectral ranges of mid-wave (MWIR) and long-wave (LWIR) infrared. The main objective of the study was to achieve the early detection of the diseases caused by the inoculated pathogens by means of the proposed measurement approaches in the two spectral ranges along a regular time course. To the best of our knowledge, this is the first time that such measurement approaches have been employed for the detection of soil-borne disease on wild rocket. The results obtained open up the possibility of developing new imaging systems for the early detection of these diseases, which can also be performed remotely and in the field.

## 2. Results

**Figure 1:**
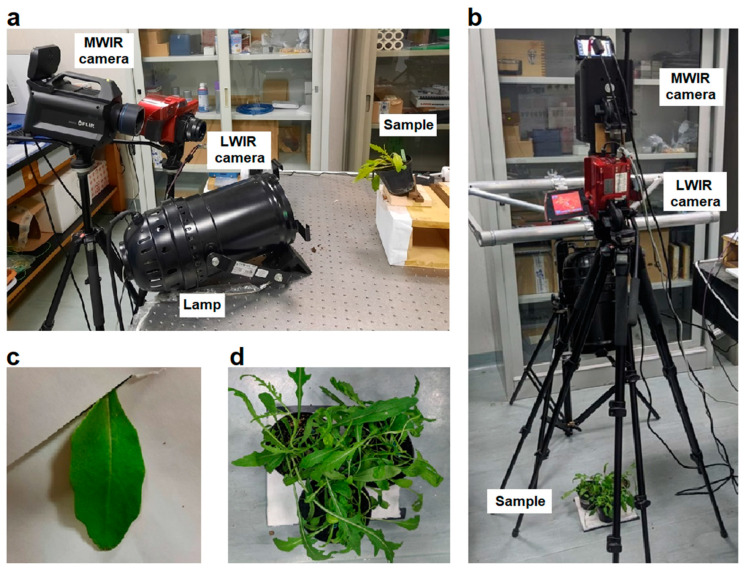
Experimental set-ups used for thermal monitoring of the plants: (**a**) AT set-up, (**b**) PT set-up, (**c**) example of leaf ready for AT analysis, and (**d**) example of a group of plants ready for PT analysis.

Herein, we present experimental measurements performed using both the active thermography (AT) and passive thermography (PT) methods to investigate the real possibility of an early detection of soil-borne diseases in wild rocket salad. Basically, AT examines sequences of infrared images of the leaves under investigation recorded during or after the application of a thermal stimulation achieved by an external excitation source (a halogen lamp). Conversely, PT is based on the analysis of single thermographic images of the leaves recorded under the natural conditions of acclimatization to the laboratory environment and without the application of any external stimulus. Using these two approaches, the thermal responses of the leaves of plants inoculated with *S. sclerotiorum* on the leaves (T1), *S. sclerotiorum* on the collars (T2), *R. solani* on the leaves (T3), *R. solani* on the collars (T4), and the leaves of non-inoculated plants (T5) were studied and compared. Figure 1 shows the experimental set-ups used for the thermal monitoring of the plants using both the active (Figure 1a) and passive (Figure 1b) approaches.

**Figure 2:**
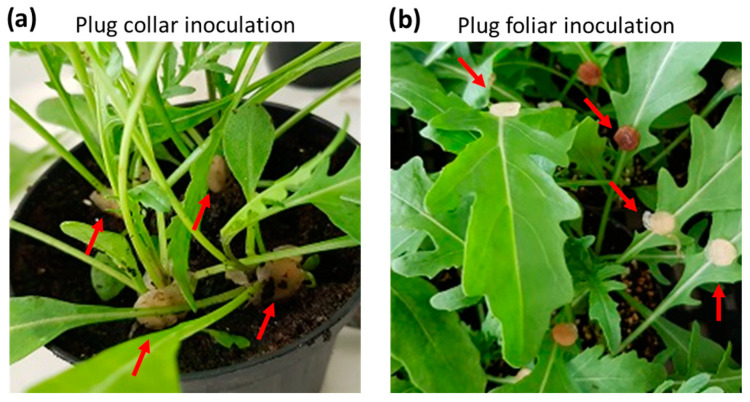
Examples of plants subjected to two artificial inoculation methods by placing mycelium plugs with *R. solani* or *S. sclerotiorum* mycelia (indicated by red arrows) on the collar (collar inoculation) (**a**) or at the petiole end of the leaf (foliar inoculation) (**b**).

Wild rocket plants subjected to artificial plug inoculations became infected regularly and developed symptoms typical of fungal disease that significantly affected the condition of the plant canopy. Figure 2 reports examples of plug inoculation on the collar (Figure 2a) and the leaf petiole end (Figure 2b). The analysis performed with both methods on the T1–T5 treatments is described below.

### 2.1. Infrared Imaging: Active Approach

Selected leaves of wild rocket plants inoculated with the soil-borne pathogens considered were thermally stimulated as described in Section 4.2, and their response was compared with that obtained on the leaves of the healthy control plants. Measurements on each single leaf (Figure 1c) were repeated daily for one week, and contextually, the leaves were visually inspected to verify the presence of symptomatic manifestations of the pathogens.

**Figure 3:**
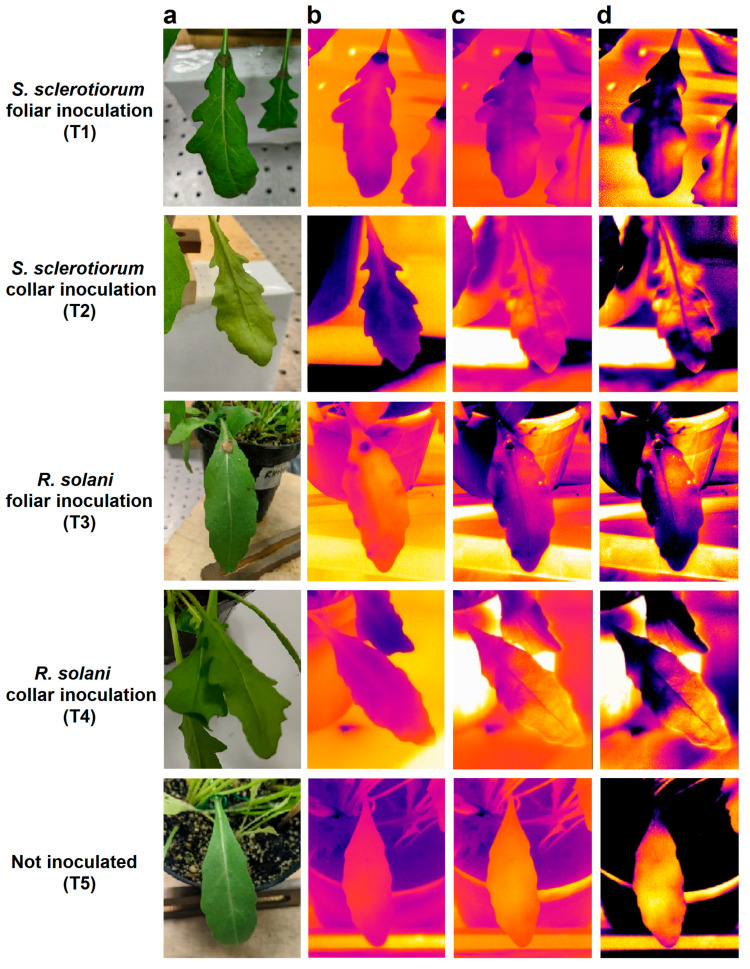
Analysis of five wild rocket leaves, one for each type of treatment taken into account, 48 h after inoculation: (**a**) visible images, (**b**) thermographic passive images recorded before heating with lamp, (**c**) thermographic active images recorded 5 s after the lamp was switched off, and (**d**) post-processing elaboration of the latter obtained using the Advanced Plateau Equalization algorithm (APE) of the FLIR System. Thermal images were acquired with an MWIR camera.

In order to illustrate and explain the phenomenon observed in the investigation, Figure 3 shows the images obtained from the analysis of five representative wild rocket leaves, one for each type of treatment considered, carried out 48 h after the inoculations: visible images (Figure 3a), thermographic passive images recorded before heating with a lamp (Figure 3b), thermographic active images recorded 5 s after the lamp was switched off (Figure 3c), and a post-processing elaboration of the latter obtained using the Advanced Plateau Equalization algorithm (APE) of the FLIR System (Figure 3d). The thermal images shown in Figure 3 and all the data discussed in this section refer to analyses made with an MWIR camera with the characteristics reported in Section 4.2.

As can be observed, in the case of the inoculations (T1–T4), both the active images (Figure 3c) and, even more evidently, the elaborated images (Figure 3d), show a spatial thermal distribution of the leaves with the presence of asymmetric anomalous areas with a different thermal response from the rest of the leaf (in purple-black colors in Figure 3c,d).

It is interesting to note from the respective visible images (Figure 3a) that no sign, lesion, or wilting is observable in correspondence with the anomalous areas identified in the active thermal images. Furthermore, these anomalous areas are not visible either in the thermal images of the non-inoculated leaf (T5) or in the passive images (Figure 3b) recorded in a thermal equilibrium condition between the leaves and the environment. This last observation highlights the importance of carrying out an active analysis to detect their presence, which is probably linked to the structural diversity of these areas of the leaf.

**Figure 4:**
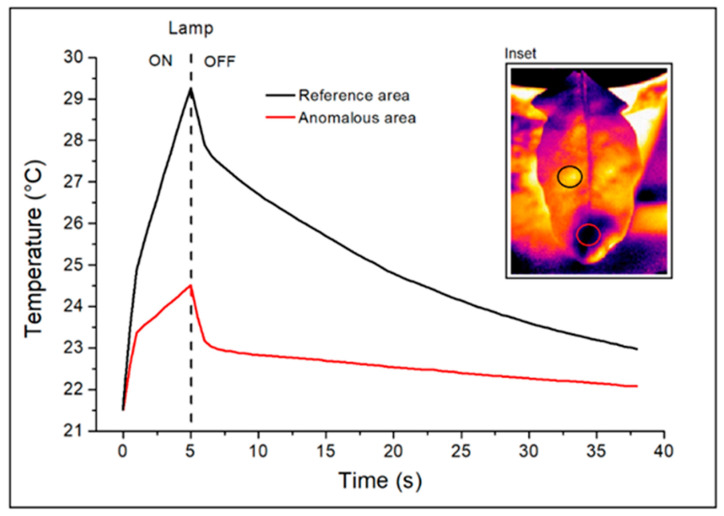
Thermal responses measured on the leaf shown in the inset: average temperature (black line) of a reference area (black circle) and average temperature (red line) of an anomalous area (red circle).

In order to investigate the nature of these areas, an example of the characteristic thermal responses detected before (t = 0 s), during (0 s < t ≤ 5 s), and after (t > 5 s) thermal heating with a halogen lamp in correspondence with both a reference area (black circle) and an anomalous one (red circle) identified on the surface of the leaf shown in the inset is reported in Figure 4. In particular, the graph refers to the temporal trend of the average temperature measured in the two areas taken into consideration.

As can be seen from the graph, the starting average temperature (at t = 0 s) was 21.4 °C for both areas. At the end of the application of the thermal stimulus (at t = 5 s), the reference area showed an average temperature of 29.4 °C and an anomalous one of 24.5 °C. Therefore, this last area shows a lower increase in temperature and a greater thermal inertia to the applied external stimulus than the reference. For this reason, during the heat recovery phase, it appears colder than the remaining parts of the leaf. The greater thermal inertia also affects the functional trend of the anomalous area after heating. In particular, this trend appears to be linear and substantially different from that shown by the reference area of the leaf, which is characterized instead, in the recovery phase, by an exponential (decreasing) trend. Most importantly, these cold areas were detected without distinction (with similar thermal and functional properties) on the leaves subjected to inoculation with the two pathogens considered, *R. solani* and *S. sclerotiorum*, both on the surfaces of the leaves and on the collars, while they were not visible in the active images recorded for the uninoculated control leaves (some examples are reported in the Supplementary File, Figure S1). These latest remarks lead to associating their presence in the inoculated ones with a response mechanism generated by the plant to the pathogens considered. Additional visible and thermographic images of inoculated wild rocket leaves are reported in Figures S2–S5.

For predictive purposes, in the AT analysis, a leaf was considered positive for the infection when, during the thermal recovery phase, it showed one or more areas characterized by average temperatures at least 0.6 °C lower than the other parts of the same leaf (Δ*T_A_* ≤ 0.6 °C). The threshold of 0.6 °C was chosen taking into consideration the natural distribution of the temperatures measured for the control leaves (not inoculated), which showed a variance in the range of 0.2–0.6 °C under the same experimental conditions. All positive results of the AT analysis occurred strictly within 96 h post inoculation (h.p.i.). In Table 1, the percentage of positive leaves detected in the various time ranges indicated and the total percentage of both positive and negative leaves for each of the five treatments investigated are reported. Obviously, the percentages of negative leaves in the various time ranges considered represent the complementary values to those shown in the table.

**Figure 5:**
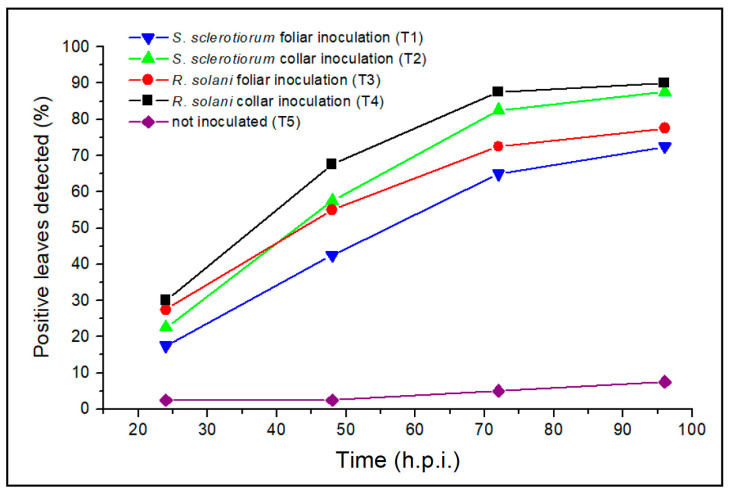
Cumulative percentage trends of leaves detected as positive for the 5 treatments at issue: *S. sclerotiorum* foliar inoculation (T1, blue line), *S. sclerotiorum* collar inoculation (T2, green line), *R. solani* foliar inoculation (T3, red line), *R. solani* collar inoculation (T4, black line), and not inoculated plants (T5, purple line).

It should be noted that all the inoculated leaves showed visible effects of the pathogens’ attacks after 6–8 days from the inoculations. Therefore, taking into consideration the data reported in Table 1, for treatments T1-T4, the percentages of positive leaves refer to true positives (TP) and the percentages of negative leaves to false negatives (FN); for treatment T5, the percentages of positive leaves refer to false positives (FP) and the percentages of negative leaves to true negatives (TN). Figure 5 shows the graph of the cumulative percentages of leaves detected as positive for the five treatments.

From the data reported in Table 1 and represented in Figure 5, notable observations can be made. TP ranged from 72.5% to 90%, TN was 92.5% (Table 1), and in general, the right previsions (RP%) of the AT analysis were 84% (calculation in the Supplementary Data). In the case of the plants inoculated with both *R. solani* and *S. sclerotiorum*, the percentage of detected positive leaves was higher for the inoculations on the collars (blue and red lines in Figure 5) than on the leaves (green and black lines in Figure 5). For both types of inoculation on the leaves and on the collars, the positivity to the AT investigation was found earlier in the case of *R. solani* (black and red lines in Figure 4) than *S. sclerotiorum* (blue and green lines in Figure 5). The plants inoculated with R. solani (both on the leaves and the collars) showed the highest percentage of positive leaves in the first and second days after inoculation (range 0–48 h.p.i.), while the plants inoculated with *S. sclerotiorum* showed the highest percentage on the second and third days (range 24–72 h.p.i.) after inoculation (Table 1).

The thermal variations ΔT_Ai_ (with *i* = 1, 2, 3, 4) with regard to the inoculated plants (T1–T4) and ΔT_A-ref_ with regard to those not inoculated (T5) were calculated at times of 24, 48, 72, and 96 h.p.i., as reported in Section 4.3, and compared by statistical *t*-test analysis. The dataset achieved showed a significant difference (*p* < 0.005) for all four inoculated treatments (T1–T4) compared to the non-inoculated one (T5) for times t ≥ 48 h.p.i.

### 2.2. Infrared Imaging: Passive Approach

The five treatments of wild rocket plants in question were also monitored through the use of the PT technique using the set-up shown in Figure 1b. In the measurements, the plants of each single treatment examined were placed in groups of three in order to simulate a cultivated bed (Figure 1d). A thermal image of each group considered in passive mode was then recorded under the natural conditions of acclimatization of the plants to the laboratory environment. Subsequently, 20 leaves were selected from the acquired thermographic image, and their overall average temperature was estimated. Figure 1d shows an example of a group of plants analyzed.

**Figure 6:**
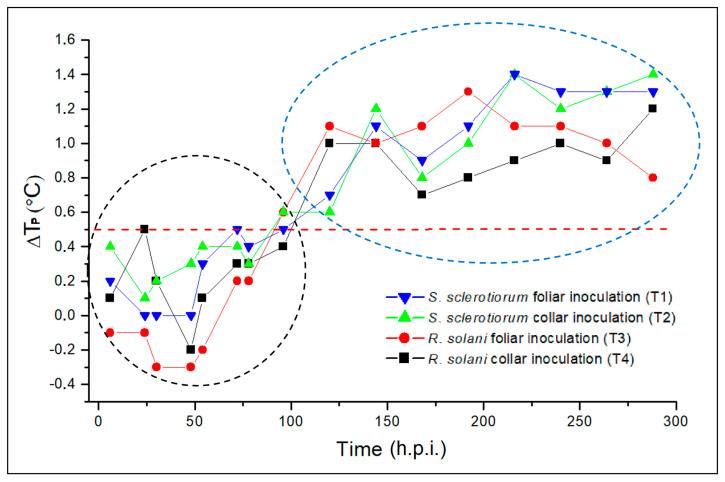
ΔT_P_ patterns measured on the inoculated plants within the monitoring period: *S. sclerotiorum* foliar inoculation (T1, blue line), *S. sclerotiorum* collar inoculation (T2, green line), *R. solani foliar* inoculation (T3, red line), and *R. solani* collar inoculation (T4, black line).

This monitoring was repeated on each group of plants twice a day for 12 days. For each group investigated, comparing the temperatures measured for the inoculated plants of the T1–T4 treatments with those obtained for the non-inoculated ones (T5), the parameter ΔT_P_ (described in Section 4.3) was calculated for each measurement performed. Figure 6 shows the ΔT_P_ trends for the treatments of the inoculated plants in relation to the monitoring period.

As can be seen from the graph, considering 0.5 °C as a threshold value, the ΔT_P_ measured can be included in two distinct clusters: a first cluster (dashed black circle) regarding the time interval 0–96 h.p.i. that includes ΔT_P_ with a maximum value of 0.5 °C, and a second cluster (dashed blue circle) regarding a time higher than 96 h.p.i. that includes ΔT_P_ with a value higher than 0.5 °C. In particular, the values in this second cluster, in the range 0.6–1.4 °C, highlight a clear and evident difference in the thermal behavior of the inoculated plants and the control ones. The positive ΔT_P_ present in this cluster indicates higher average temperatures for the inoculated groups than those obtained for the control group. The latter evidence can be associated with the presence of pathogens, whose action progressively reduces the local water content present in the leaves of the inoculated plants.

### 2.3. Monitoring through Visible Inspection

**Figure 7:**
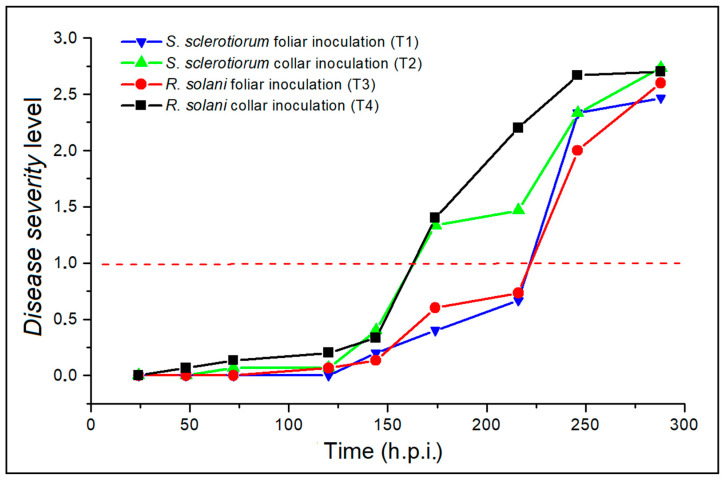
Trend of the average disease severity level used to assign the state of health to plants by visual analysis in the case of the four treatments considered: *S. sclerotiorum* foliar inoculation (T1, blue line), *S. sclerotiorum* collar inoculation (T2, green line), *R. solani* foliar inoculation (T3, red line), and *R. solani* collar inoculation (T4, black line).

During the course of the disease, the plants investigated were monitored by visual inspection to assess their state of health over time by evaluating the average severity level of the overall detrimental effects on the canopy. Both the pathogens started infection within 24 h.p.i., with the occurrence of soft, water-soaked spots on the surface of the leaf or at the collar covered by the plug and directly in contact with the fungal mycelium. Thus, the pathogenesis continued over the next 48 h by slowly widening the hydropic halos in the area surrounding the point of infection by a few millimeters. Progressively, spots evolved in the typical soft and dry parenchymatic rots caused by *S. sclerotiorum* and *R. solani* attacks, respectively, without inducing visually appreciable macroscopic effects on the canopy not directly in contact with the pathogens until, on average, the sixth day. Indeed, as time progressed and the vascular apparatus of the collar or the petiole became more affected, the vegetative state of the plant canopy increasingly reflected the damaging indirect effects of the disease. Figure 7 shows the trend with respect to the h.p.i. of the estimated average level for the four types of treatments considered.

**Figure 8:**
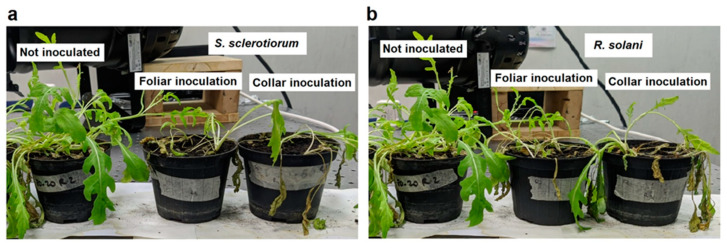
Comparison between non-inoculated and inoculated plants 10 days after the inoculations: (**a**) non-inoculated plant compared with plants inoculated with *S. sclerotiorum* (both foliar and collar inoculation), and (**b**) non-inoculated plant compared with plants inoculated with *R. solani* (both foliar and collar inoculation).

Although the infection’s course under *R. solani* and *S. sclerotiorum* inoculum was significantly faster in the cases of the collar attacks and leaf infections, respectively, distinguishable wilting effects on the canopy were not observed before 120 h.p.i. under both pathogens. In detail, as can be seen from the graph in Figure 7, the disease status curves indicate that *R. solani* proved to be the more aggressive pathogen, starting to wilt plants just at 120 h.p.i., compared to *S. sclerotiorum*, which, on the other hand, exhibited even a one-day delay in showing the same degree of damage. As a result, the plants finally fell under the action of the collar dry rot agent 8 days after inoculation; the following day, the maximum degree of disease was also observed for the remaining treatments. Furthermore, in the case of both *R. solani* and *S. sclerotiorum* pathogens, the plants with collar inoculations reached level 1 (dashed red line), associated with the onset of the pathological state, after a time greater than 150 h.p.i., while the plants with foliar inoculations inoculated after a time greater than 220 h.p.i. As an example, Figure 8 shows visible photos in which some of the monitored inoculated plants are compared with the non-inoculated reference control. The photos refer to 10 days after the inoculations.

### 2.4. Comparison of MWIR and LWIR Analyses

**Figure 9:**
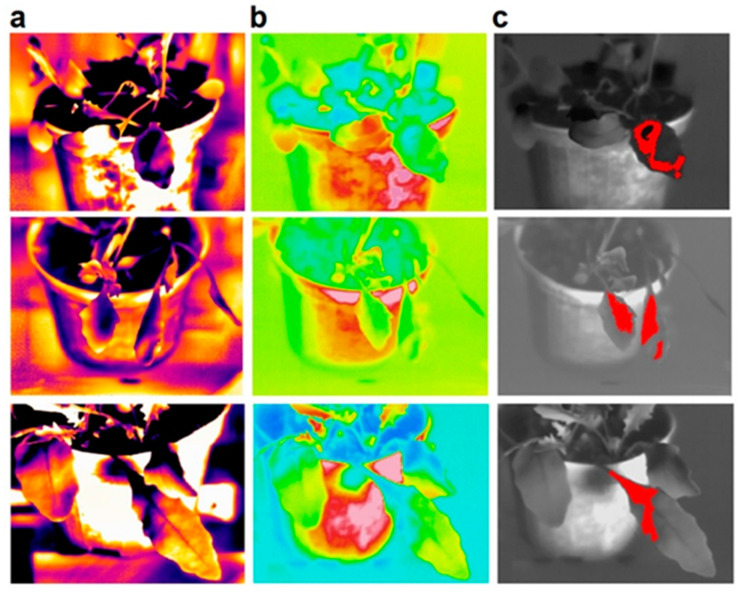
Comparison of thermographic images obtained for some of the investigated leaves: images achieved with the MWIR camera (**a**), images achieved with the LWIR camera (**b**), and a post-processed version of the latter (**c**).

The potential application of the thermographic techniques shown in this work was investigated from a technological point of view by carrying out comparative tests analyzing the inoculated plants using an MWIR camera with a cooled InSb sensor (to which all the measurements reported up to now in this work refer) and an LWIR camera with an uncooled microbolometric VOX sensor and other characteristics described in Section 4.2. As an example, Figure 9 shows some thermal images of inoculated leaves recorded with the MWIR camera (a) and the LWIR camera (b).

As can be seen by comparing the images in Figure 9, in the case of the LWIR images, there is an evident and clear reduction in the visibility of the anomalous areas (black-purple colors in the MWIR images) on the leaves analyzed. This is mainly due to the lower technical characteristics of the LWIR camera compared to the MWIR one. In particular, the lower thermal and spatial resolution and the greater thermal/electrical noise to which the images recorded with the LWIR camera are exposed make the anomalous areas not always clearly appreciable, especially in the case of ΔT at the limit (0.4–0.6 °C) of the values considered symptomatic in the method introduced and used in the previous sections. However, the visibility and detection of many of the anomalous areas found on the leaves can certainly be improved by carrying out appropriate post-processing based, for example, on an adequate setting of the temperature range and of the color bar, as shown by the images in Column (c).

## 3. Discussion

The digital detection of plant diseases can help optimize management tactics that rely on an increasingly limited number of available means to fight pathogenic fungal infections. Wild rocket cultivation systems are subjected to high technology at many stages of the production cycle, from cultivation to post-harvesting, up to the opening of the packages ready for consumption. Very recent studies have highlighted the potential of optoelectronic hyperspectral technologies to achieve automatic, non-destructive, and rapid early detection of the major soil-borne fungal diseases of wild rocket and other baby leafy vegetables for the fourth range supply chain, revealing changes in the biochemical information [7,30,31,32].

Supported by visual inspection, robust sampling methods and reliable molecular laboratory analysis (e.g., qPCR) to identify fungi isolated from samples of putatively diseased plants are the most currently available methodologies to monitor and diagnose soil-borne fungal diseases on wild rocket and/or other similar baby-leaf crops, respectively [33,34]. Real-time PCR protocols are limited for direct diagnosis on infected plant samples by the difficulty of having suitable probes [35,36], whereas for rapid in planta detection of the target pathogens, loop-mediated isothermal amplification (LAMP) is being developed against both *S. sclerotiorum* [37] and *R. solani* [38]. Molecular detection techniques, however, are time-, reagent-, and labor-intensive and can provide only limited qualitative information on ongoing infections compared to image-based detection tools.

In this study, thermography was explored as a means to monitor wild rocket crop disease events, support decision-making processes through the early detection of outbreaks, and guide phytosanitary remediation in space and time. Thermal imaging is based on the acquisition of the electromagnetic spectrum emitted by plants in the infrared region, returning the spatial distribution of the surface thermographic temperature represented by an image. Stress factors (e.g., water stress, plant diseases, etc.) that affect both the ability of plants to emit energy and the leaf temperatures can be monitored by detecting changes in these thermographic images.

In this work, AT and PT were applied to scan the response of potted wild rocket plants to the controlled infections with *R. solani* and *S. sclerotiorum*, which showed, at visual assessment, an initial overall appearance of symptoms on the canopy after 150 h.p.i., on average. The dynamics of fungal attacks registered under the experimental conditions were consistent with what normally occurs in the field in the case of natural pressure from the pathogens, with a progressive decline in the vegetative state of the plants [39]. It is interesting to note that, for all infections, the ΔT_P_ patterns calculated on the PT acquisitions were similar in shape to that of the average severity level established by the visual assessment, albeit with a temporal advance of the increase towards higher levels. PT analysis detected disease between 72 and 120 h.p.i., with values of ΔT_P_ between inoculated/healthy plants higher than 0.5 °C. This time interval was considerably longer than the prediction times (range 24–72 h.p.i.) obtained with the AT analysis. Interestingly, the response of the sampled plants associated with controlled halogen-lamp thermal stimulation showed an asymmetrical spatial distribution of leaf temperature during the thermal recovery phase.

The current thermographic investigation identified useful indicators of soil-borne disease occurrence, such as the presence of abnormal areas on the leaf surface or their mean temperature, which can be linked to the action of the considered pathogens. As a matter of fact, Cao et al. [40] also found, through the PT approach, a temperature spatial shift along the Sclerotinia rotting progression line on rapeseed leaves within 1.7 °C from the necrotic to the pre-infected area, assuming an altered metabolism due to the production of salicylic acid in the area, underlying the phenomenon. Reasonably, the higher thermal inertia of the anomalous areas could be associated with the presence of a higher local water content in these areas [41], effectively due to the formation of water-soaked lesions caused by cell breaking by pathogen pectolytic enzymes [42] and/or alterations in leaf transpiration [43]. An increase in the local water content in plant leaves as a response mechanism to abiotic stress was observed during the real-time monitoring of plants subjected to artificial UV-B irradiation [21]. Actually, the link between thermographic signals and plant water status was observed on wild rocket with the continuation of the drought conditions [44]. Meanwhile, on young lettuce plants, thermographic crop water stress indices, calculated by combining leaf temperatures with dry and wet reference temperatures, were used to classify Rhizoctonia basal rot [45]. Over the long distance from the point where the pathogen entered the host, the thermographic appearance of wild rocket leaves may have depended on changes in the ability to dissipate heat. Leaf cooling due to evapotranspiration is regulated by stomatal conductance, which modulates the rate of gas exchange [46]. Conversely, the effectors that induce the opening/closing of stomata can be traced by thermographically measuring decreasing/increasing leaf temperatures. As a consequence of a pathogenic attack, the stimulation of the plant immune response, that moves systemic signals emerging from the infected cells to those nearby [47,48], may induce the regulation of stomatal movements in the canopy [49,50]. In this study, AT indicated a perturbation in the heat distribution on the leaves of diseased plants from the earliest hours of the pathogenesis. These findings confirm the potential of thermal imaging in making an early detection of soil-borne diseases in wild rocket, almost 3–6 days before the macroscopic involvement of the whole canopy, which would allow the detection of outbreaks during a cursory visual inspection. Specifically, for plants inoculated with *R. solani* (both on the leaves and the collars), the detection occurred within 0–48 h.p.i., while for the ones inoculated with *S. sclerotiorum*, within 24–72 h.p.i. *R. solani* proved to be more pervasive, being able to trigger a response in the plant, as captured by thermography, in an earlier and narrower timeframe, confirming its particular aggressiveness [51].

On the other hand, the results of the comparison of the analyses in the two infrared band ranges showed that thermographic instruments for the early detection of plants infected with soil pathogens can also be developed with LWIR bolometer cameras. Due to their commonly lower cost compared to MWIR technologies, these instruments can also be more attractive from a commercial point of view. Obviously, the lower technical performance of LWIR cameras compared to MWIR cameras, especially in terms of spatial, temporal, and thermal resolution, partially affects the monitoring results, leading to a loss both in terms of the percentage of positive leaves detected and in the prediction times calculated from inoculation.

Both of these feared diseases are the subject of much attention by phytopathologists in these crops in efforts to improve the preventive control protocols and reduce the exacerbation of soil sickness phenomena. Digital tools, such as emerging thermography, may support disease management in wild rocket cropping systems, although with non-specific fingerprinting.

## 4. Materials and Methods

### 4.1. Plant Growth and Inoculation Methods

The soil-borne fungal pathogens used in this study were *R. solani* (RS) AG-4 and *S. sclerotiorum* (SS), taken from the microbial collection of CREA-Centro di Ricerca Orticoltura e Florovivaismo (Pontecagnano Faiano, Italy), and previously tested for pathogenicity on wild rocket [7]. Fungi were maintained on potato dextrose agar (PDA, Oxoid) slants at 4 °C until use. The experiments were carried out on 1-month-old wild rocket (*D. tenuifolia*) cv. Tricia (Enza Zaden, Italy), grown (5 plants pot^−1^) in plastic pots (90 mm diameter) on autoclaved peat-based substrate (Klasmann-Deilmann GmbH, Geeste, Germany), arranged in a nursery glasshouse. Then, pots were transferred into a mini-lab plastic greenhouse, and plants were subjected to artificial infection by placing mycelium plugs (0.5 cm in diameter), separately at the petiolar end of each leaf (foliar inoculation, Figure 2a) or at the collar of each plant (collar inoculation, Figure 2b). Non-inoculated pots were used as reference controls. Then, inoculated plants were kept in the plastic chamber, microsprayed with sterile water to maintain > 98% relative humidity and incubated at room temperature (25 ± 2 °C) in a completely randomized scheme. Over the next 14 days, the plants were subjected to thermographic scans (see Section 4.2) and visual assessment of the symptomatic evolution, assigning a degree of severity to each plant in the pot according to a 0–3 rating scale (0: healthy; 1, 2, and 3: diseased at early, moderate, and severe degree, respectively) [30]. Daily, each plant per pot inoculated with *R. solani* and *S. sclerotiorum* was assigned one of the levels indicated in Table 2 after an accurate visual analysis. The average value for the levels was estimated by visual analysis of five plants for each treatment considered.

The experimental design included five pots for each of the two inoculation methods indicated above and one non-treated control, each containing five plants for a total of seventy-five plants. Four experiments were performed consecutively.

### 4.2. Infrared Imaging Measurements

#### 4.2.1. Methods: Active and Passive Thermographic Approaches

Wild rocket plants suitably inoculated with the soil-borne pathogens and those not inoculated (controls) were analyzed using IR imaging techniques. Two different analysis approaches, active and passive, were performed as described below:

Active Thermography (AT): Leaves of the treatments examined were individually thermally stimulated by means of a lamp, and the thermal response obtained after the stimulation (thermal recovery phase) was recorded and analyzed for each of them. For each leaf, the measurement was repeated 1–2 times a day during the whole monitoring period.

Passive Thermography (PT): For each of the treatments examined, groups of plant pots were analyzed recording single thermographic images in passive mode (i.e., without the application of any external stimulus) under the natural conditions of acclimatization to the laboratory environment. For each group of plants, the measurement was repeated 2 times a day during the whole monitoring period.

#### 4.2.2. Set-Up and Instrumentation

The measurements were carried out using infrared cameras operating in 2 spectral ranges: MWIR and LWIR.

The MWIR camera was a FLIR X6580 sc with a cooled indium antimonide (InSb) sensor, spectral range 3.5–5 μm, FPA 640 × 512 pixels, and NETD ~20 mK at 25 °C. The measurements were carried out using a germanium objective with a focal length of 50 mm and an IFOV of 0.3 mrad. The recordings with this camera were made at a frame rate of 5 Hz, and the commercial ResearchIR (FLIR Systems Inc, Wilsonville, Oregon, USA) software was used to monitor the temperature obtained on the leaves in real time and for the basic analysis operations.

The LWIR camera was an AVIO TVS500 with an uncooled microbolometric VOX sensor, spectral range 8–14 μm, FPA 320 × 240 pixels, and NETD ~50 mK at 25 °C. The measurements were carried out using a germanium objective with a focal length of 22 mm and an IFOV of 1.07 mrad. The recordings with this camera were made at a frame rate of 5 Hz, and the commercial software IRT Analyzer (GRAYESS) was used to monitor the temperature obtained on the samples in real time and for the basic analysis operations.

For the active approach, each leaf was stimulated by means of a thermal impulse of 5 s carried out with the use of a halogen lamp (maximum power 1 kW) positioned approximately parallel to the normal of the investigated leaf surface (with a maximum inclination of 5°) and connected to a control system that allowed to appropriately select the on/off duration and the power of the pulse. The latter parameter was appropriately selected in the various measurements carried out to induce an increase in temperature between 3 and 8 °C on the leaves.

During the measurements, the thermo-hygrometric data of the laboratory were monitored. The data showed values between 20 and 23 °C for the temperature and 48–55% for the humidity.

### 4.3. Statistical Analysis and ΔT Parameters

The experimental activity took place during 4 replications of the measurements in the laboratory in order to collect sufficient data for a statistical analysis. These measurements were focused on the detection of the effects achieved on plants inoculated with *R. solani* and *S. sclerotiorum* on two different organs: leaves and collars. During these analyses, the thermal response of leaves of plants inoculated with *S. sclerotiorum* on leaf (T1), *S. sclerotiorum* at collar (T2), *R. solani* on leaf (T3), *R. solani* at collar (T4), and non-inoculated (control) plants (T5) was monitored daily for 7–14 days using both AT and PT approaches described in Section 4.2.

In the case of AT analysis, for 40 leaves from each treatment (T1, T2, T3, and T4), the values ΔT_Ai_ = T_COL_ − T_REF_ (with *i* = 1, 2, 3, 4) were calculated, where T_COL_ and T_REF_ represent the average temperature of an anomalous cold area and a reference one detected on the surface of the leaves, respectively. In the case of non-inoculated plants (T5), the maximum temperature difference measured on the surface of the leaves was considered to be ΔT_A-ref_ = ΔT_MAX_.

Normality of distributions ΔT_Ai_ and ΔT_A-ref_ achieved at 24, 48, 72, and 96 h.p.i was verified by Shapiro–Wilk test and analyzing kurtosis and skewness. Individually, a *t*-test was conducted for mean comparison between the datasets ΔT_Ai_ and ΔT_A-ref_ for *i* = 1, 2, 3, 4 and for each time considered. Statistical parameters were calculated in a Microsoft Excel spreadsheet using its internal functions.

In the case of PT analysis, for groups of three plants from each treatment, the values ΔT_Pi_ = T_INO_-T_CON_ (with *i* = 1, 2, 3, 4) were calculated, where *T_INO_* and *T_CON_* represent the average temperatures detected on the group of inoculated plants (T1–T4) and that on the control group (T5), respectively. Both T_INO_ and T_CON_ were evaluated by averaging the temperatures detected on 20 leaves of the group. ΔT_Pi_ values were calculated for each time of measurement, and their trend is graphicated in Figure 6.

In this last case, since it was considered a dataset achieved at a different time and built directly on the thermal variations between inoculated and non-inoculated plants (ΔT_Pi_), it was not possible to conduct any statistical comparison.

## 5. Conclusions

In this work, the AT and PT techniques were employed and tested to monitor the thermal and physiological status of plants inoculated with soil-borne pathogens *R. solani* and *S. sclerotiorum.* The analyses were performed using infrared cameras in two different spectral ranges, MWIR and LWIR. For both types of pathogens, the performance for an early diagnosis through IT-based measurements were investigated for plant inoculated in two different regions: the collars and the leaves. The results obtained show how both the AT and PT techniques can be used to carry out an early identification of the effects of the pathogens on the investigated leaves/plants, allowing the detection of the symptoms of the disease 3–6 days before they are detectable by visual analysis. Our results confirm the potential of these techniques in monitoring wild rocket for the preventive detection of soil diseases with the aim of avoiding their extension and preserving the crops.

## Figures and Tables

**Table 1:** Percentage of wild rocket leaves detected as positive in the time ranges 0–24, 24–48, 48–72, and 72–96 h post inoculation (h.p.i.) and the total percentage of leaves assessed as both positive (True Positive, TP; False Positive, FP) and negative (False Negative, FN; True Negative, TN) from plants subjected to artificial foliar or collar inoculation with *R. solani* or *S. sclerotiorum* compared to non-infected control.

Treatments	Positive Leaves (%)				Total (%)	
	0–24 h.p.i.	24–48 h.p.i.	48–72 h.p.i.	72–96 h.p.i.	Positive	Negative
*S. sclerotiorum* foliar inoculation (T1)	17.5	25	22.5	7.5	72.5 (TP)	27.5 (FN)
*S. sclerotiorum* collar inoculation (T2)	22.5	35.0	25.0	5.0	87.5 (TP)	12.5 (FN)
*R. solani* foliar inoculation (T3)	27.5	27.5	17.5	5.0	77.5 (TP)	22.5 (FN)
*R. solani* collar inoculation (T4)	30.0	37.5	20.0	2.5	90.0 (TP)	10.0 (FN)
Not inoculated (T5)	2.5	0	2.5	2.5	7.5 (FP)	92.5 (TN)

**Table 2:** Levels used to assign disease severity status to plants by visual inspection.

Score	Overall Appearance of the Symptoms	Description
0	Not detectable	Canopy without macroscopic withering
1	Initial	Canopy slightly wilted and/or with curling leaves
2	Moderate	Plant showing leaf yellowing
3	Severe	Plant showing marked yellowing and/or wilting

## Data Availability

The data presented in this study are available in this article and the Supplementary Materials.

## Supplementary Materials

The following supporting information can be downloaded at https://www.mdpi.com/article/10.3390/plants12081615/s1, Figure S1: Examples of active thermography analysis for uninoculated control leaves of rocket plants; Figures S2–S5: Examples of active thermography analysis for leaves of rocket plants inoculated with treatments T1, T2, T3, and T4. Right Prevision (RP%) calculation.
