# Supplementary material for: Active vs. Passive Thermal Imaging for Helping the Early Detection of Soil-Borne Rot Diseases on Wild Rocket [Diplotaxis tenuifolia (L.) D.C.]
Source: Plants (Basel). 2023 Apr 11;12(8):1615. doi: 10.3390/plants12081615 (PMC10141070; doi:10.3390/plants12081615)
Supplement: Supplementary file 1 [file plants-12-01615-s001.zip › plants-2313400-supplementary.pdf]

Supplementary Data

Active Vs Passive thermal imaging for helping the early detection of soil-borne diseases on wild rocket salad crop caused by Rhizoctonia solani Kühn and Sclerotinia sclerotiorum (Lib.) de Bary

Massimo Rippa[1,*], Andrea Pasqualini[2], Rossella Curcio[1], Pasquale Mormile[1], Catello Pane[2]

[1] Institute of Applied Sciences and Intelligent System "E. Caianiello" of CNR, 80078 Pozzuoli (Na), Italy
[2] Consiglio per la Ricerca in Agricoltura e l'Analisi dell'Economia Agraria, Centro di Ricerca Orticoltura e Florovivaismo, via Cavalleggeri, 25, 84098 Pontecagnano Faiano, Italy
* Correspondence: m.rippa@isasi.cnr.it

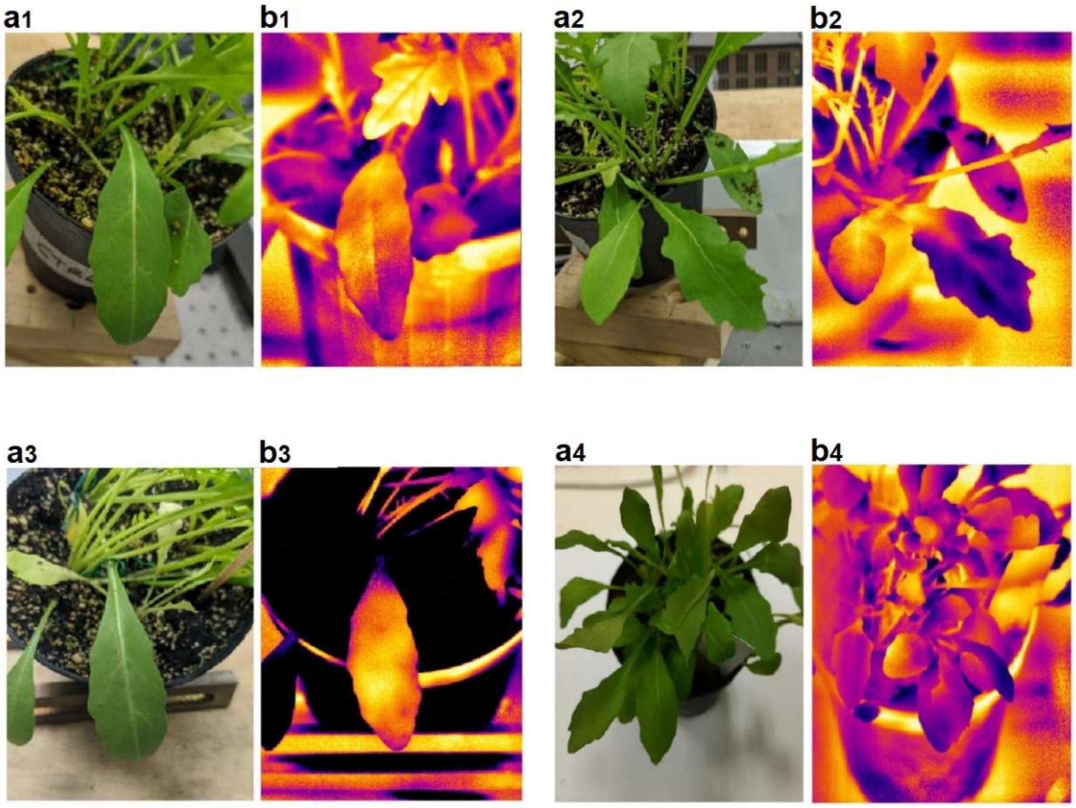

Figure S1. Examples of active thermography analysis for uninoculated control leaves of rocket plants: a) visible images and b) post-processed thermal images.

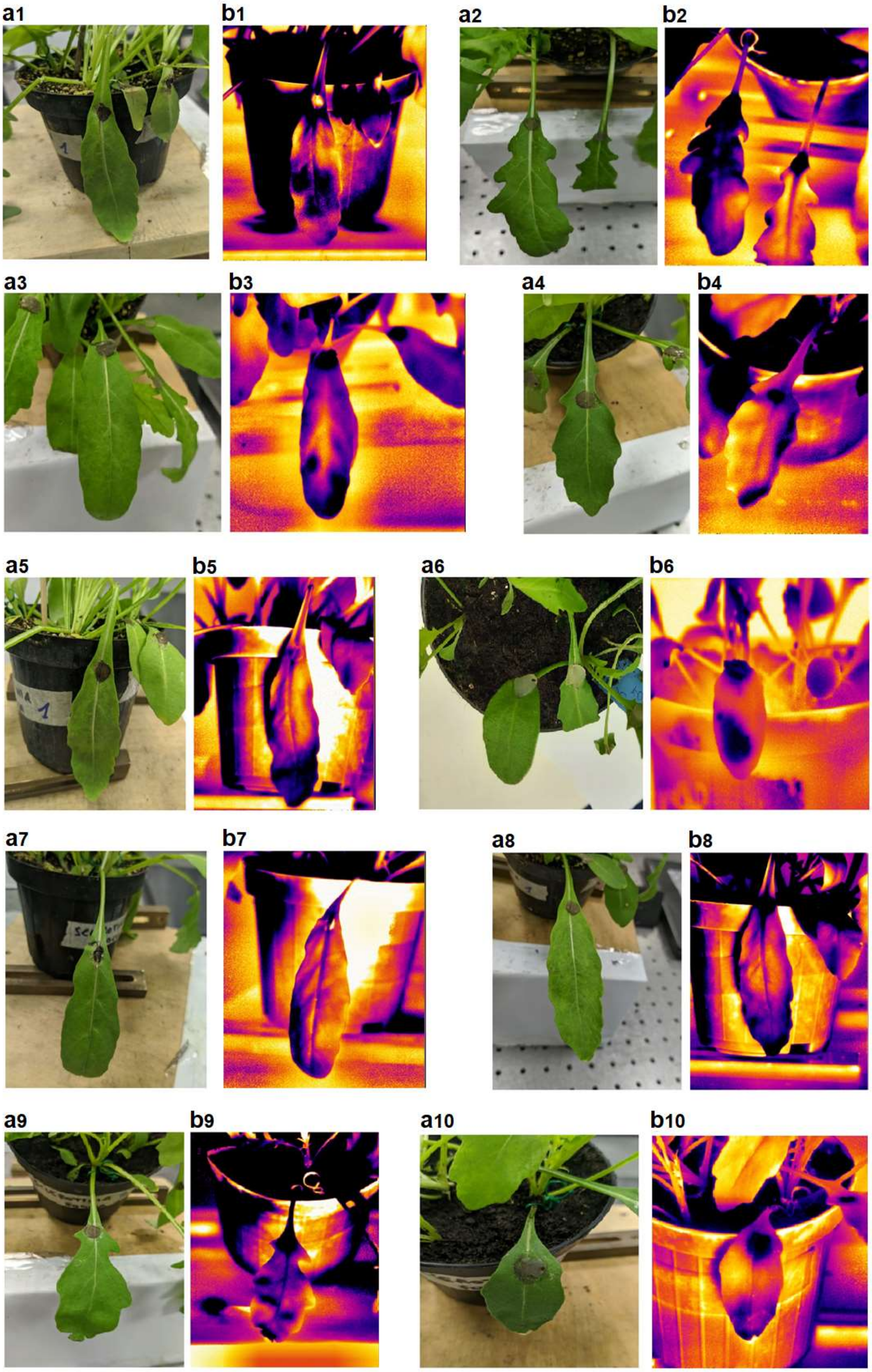

Figure S2. Examples of active thermography analysis of rocket plants inoculated with *S. sclerotiorum* on leaves (T1): a) visible images and b) post-processed thermal images.

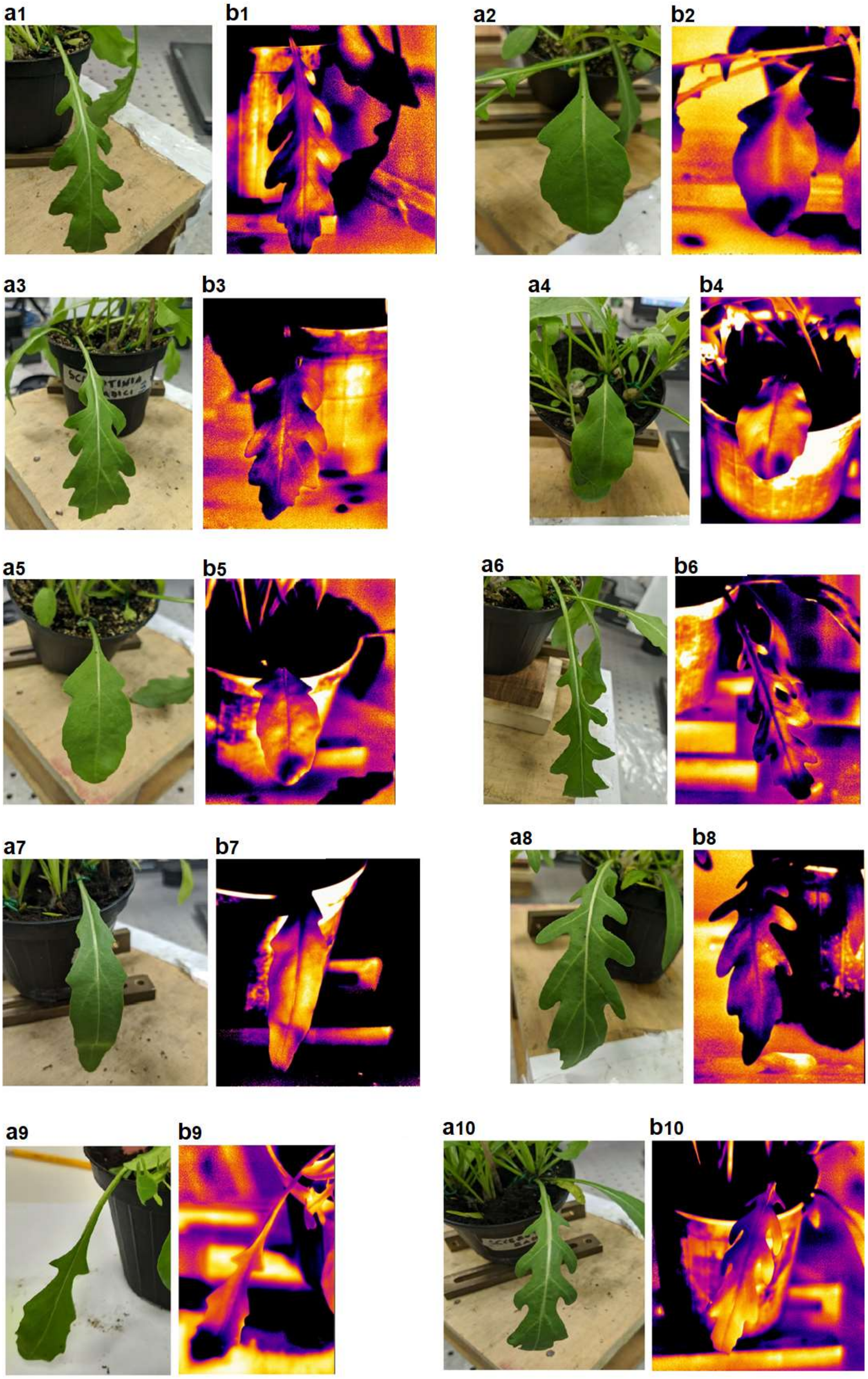

Figure S3. Examples of active thermography analysis of rocket plants inoculated with *S. sclerotiorum* on collars (T2): a) visible images and b) post-processed thermal images.

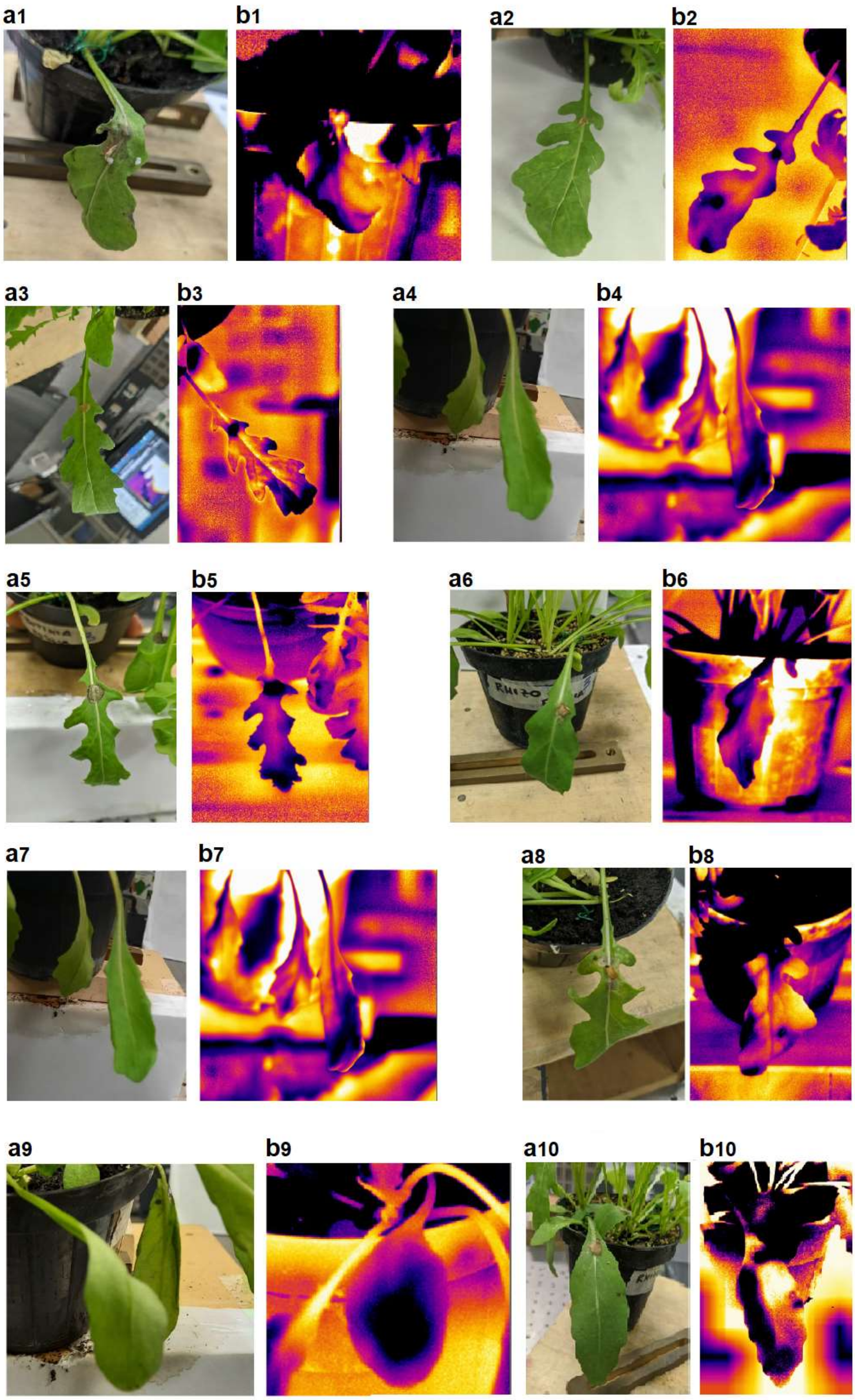

Figure S4. Examples of active thermography analysis of rocket plants inoculated with *R. solani* on leaves (T3): a) visible images and b) post-processed thermal images.

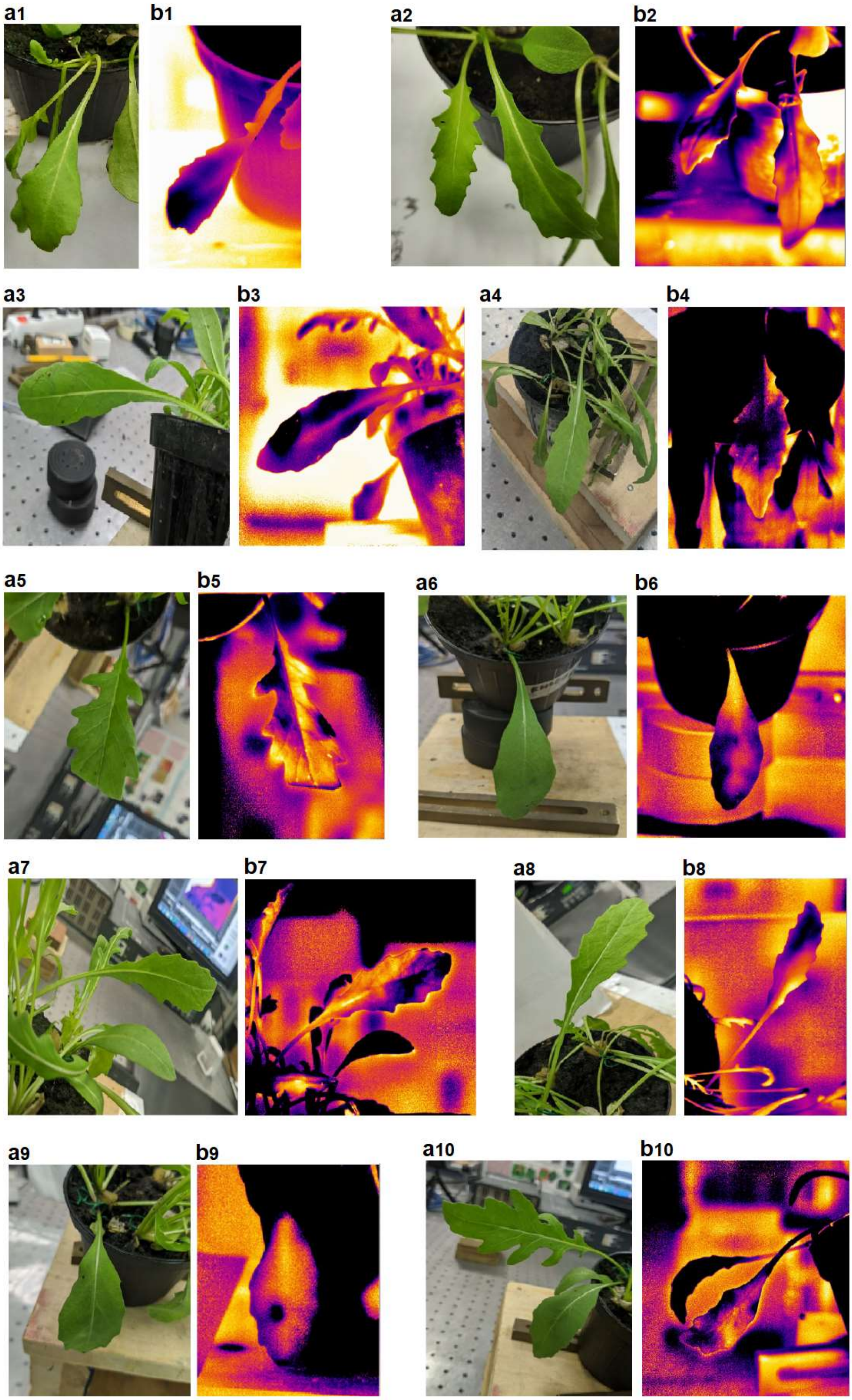

Figure S5. Examples of active thermography analysis of rocket plants inoculated with *R. solani* on collars (T4): a) visible images and b) post-processed thermal images.

Right prevision (RP%) calculation:

$$RP\% = \frac{\sum_i^4 TP_i + TN}{5} = \frac{72.5 + 87.5 + 77.5 + 90.0 + 92.5}{5} = 84$$

Where TP_i are the True Positive found in the treatments T1, T2, T3 and T4 and *TN* the True Negative found for not inoculated plants T5.
